# Bidirectional Active Piezoelectric Actuator Based on Optimized Bridge-Type Amplifier

**DOI:** 10.3390/mi12091013

**Published:** 2021-08-26

**Authors:** Weiqing Huang, Junkai Lian, Mingyang Chen, Dawei An

**Affiliations:** School of Mechanical and Electrical Engineering, Guangzhou University, Guangzhou 510006, China; meehuangweiqing@gzhu.edu.cn (W.H.); 2112007107@e.gzhu.edu.cn (J.L.); mychen0619@163.com (M.C.)

**Keywords:** piezoelectric actuator, bidirectional active drive, bridge mechanism, sensitivity analysis

## Abstract

Piezoelectric actuators based on bridge displacement amplifying mechanisms are widely used in precision driving and positioning fields. The classical bridge mechanism relies on structural flexibility to realize the return stroke, which leads to the low positioning accuracy of the actuator. In this paper, a series bridge mechanism is proposed to realize a bidirectional active drive; the return stroke is driven by a piezoelectric stack rather than by the flexibility of the structure. By analyzing the parameter sensitivity of the bridge mechanism, the series actuation of the bridge mechanism is optimized and the static and dynamic solutions are carried out by using the finite element method. Compared with the hysteresis loop of the piezoelectric stack, the displacement curve of the proposed actuator is symmetric, and the maximum nonlinear error is improved. The experimental results show that the maximum driving stroke of the actuator is 129.41 μm, and the maximum nonlinear error is 5.48%.

## 1. Introduction

Piezoelectric (PZT) actuators are widely used in ultra-precision machining and measurement, large-scale integrated circuit manufacturing, scanning probe microscopes, aerospace, micro-robots, optics, and the medical field due to their high resolution and high positioning accuracy [1,2,3,4]. Since the driving stroke of the PZT stack is only about 0.1% of its own length [5,6], many scholars have designed various types of displacement amplification mechanisms to increase the output displacement of the PZT stack [7,8,9]. For example, Sun et al. proposed a two-dimensional micro-positioning platform with equal stiffness and equal stroke based on the hourglass displacement amplification mechanism, with a displacement magnification close to 10 times [10]. Jun Hyung Kim proposed a three-dimensional series double-bridge flexure hinge mechanism, and when appropriate structural parameters were selected, the displacement magnification reached 30 times [11]. Chen et al. designed a two-stage lever mechanism using elliptical flexure hinges, with a displacement magnification of 40 times [12].

The bridge-type displacement amplification mechanism is widely used in the design of PZT actuators because of its advantages such as a high displacement magnification, a compact structure, a large bearing capacity, and machining convenience [13,14,15]. The push stroke of the classical bridge mechanism is realized by the active drive of the PZT stack, but the return stroke can only be realized by the flexible recovery of the flexible beam [16,17]. The displacement curve is asymmetric and nonlinear, which leads to the poor positioning accuracy of the actuator. In addition, the actuators of the classical bridge mechanism can be regarded as the “cantilever-beam-type”, with one end fixed and the other end with output displacement [18]. Usually, the natural frequency of such a PZT actuator with only one end fixed is low.

Establishing the hysteresis nonlinear model of the PZT stack and designing the feedforward control algorithm to reduce the nonlinear error of the output displacement of the PZT actuator is an effective method to improve the positioning accuracy of the actuator [19,20]. In addition, there is a displacement feedback control method that can achieve high positioning accuracy by detecting the output displacement signal and feedback adjusting the driving voltage, but it can only be applied to static or quasi-static positioning control [21]. In the above method, the input voltage of the PZT stack is adjusted by using feedforward or feedback signals to reduce the nonlinear error of the output displacement of the actuator. This method places a high demand on the computing power of the system.

The main objective of this study is to reduce the nonlinear error of the actuator output displacement curve and reduce the dependence on the computing power of the controller. Taking the classical bridge mechanism as the research object, a series PZT actuator is optimized and designed based on parameter sensitivity analysis. Both the push stroke and return stroke are driven by the PZT stack, which overcomes the drawback due to the classical bridge actuators relying on the flexibility of the structure to realize the return stroke, and improves the positioning accuracy of the actuators. The maximum driving stroke, maximum stress point, and resonant frequency of the actuator are analyzed by the finite element method. An experimental platform is established to test the driving stroke, hysteresis, non-driven displacement, response speed, and resonance frequency of the actuator.

## 2. Theoretical Model of the Classical Bridge Mechanism

**Figure 1:**
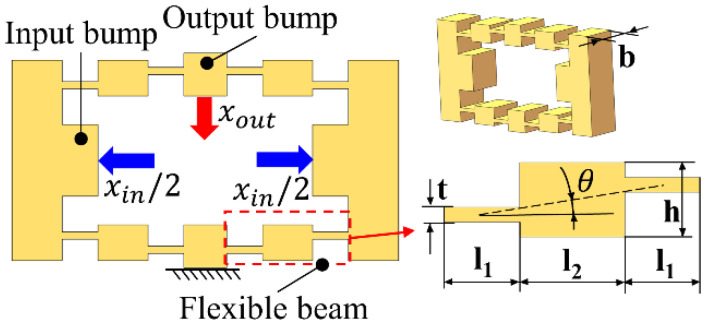
Classical bridge mechanism.

The classical bridge mechanism has bidirectional symmetry [22]. The quarter model of the bridge mechanism is usually taken as the research object to analyze its displacement output characteristics. Compared with the asymmetrical mechanism such as the lever displacement amplification mechanism, the symmetrical structure can avoid the generation of non-driven displacement in principle. The structure of the bridge mechanism is depicted in Figure 1. Four flexible beams are connected with the output bump and the input bump to form a bridge mechanism. The flexible beam can be regarded as a rectangular bar with flexible hinges at both ends. The main structural parameters of the bridge mechanism are indicated in Figure 1, including the thickness of the bridge mechanism b, the length of the flexible hinge $l_{1}$ and the thickness t, the length of the rectangular rod $l_{2}$, the thickness of the elastic rod h, and the angle θ between the geometric center connecting line and the horizontal line of the flexible hinge.

### Force Analysis of Classical Bridge Mechanism

**Figure 2:**
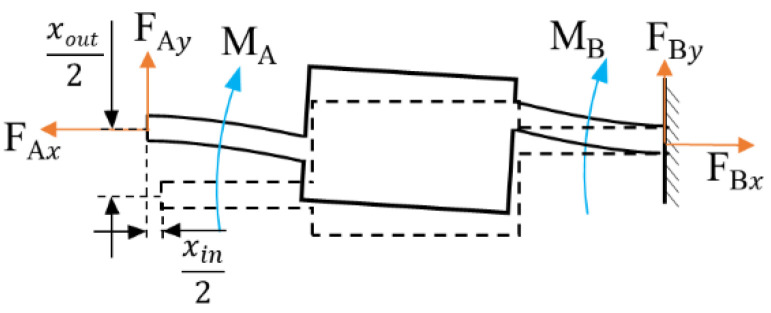
Force analysis of a flexible beam.

As shown in Figure 2, the input bump and the output bump are regarded as rigid bodies to conduct force analysis of the flexible beam. The flexible beam is subjected to the horizontal input force $F_{in}$ of the PZT stack, and the flexible beam produces input displacement $x_{in}$ and output displacement $x_{out}$.

In the working process of the bridge mechanism, the flexible beam is in the linear flexible stage with slight deformation, and the forces and moments in both the horizontal and vertical directions are in equilibrium. Then, the following relation can be obtained:

(1) $$F_{Ax}=F_{Bx}=F_{in}/2$$

where $F_{in}$ represents the input force of the bridge mechanism.

(2) $$2M_{A}=2M_{B}=F_{in}\left(l_{1}+l_{2}\right)\tan\theta /2$$

where $M_{A}$ represents the torque caused by $F_{Ax}$, $M_{B}$ represents the torque caused by $F_{Bx}$, $\left(l_{1}+l_{2}\right)\tan\theta$ represents the vertical distance between the horizontal forces $F_{Ax}$ and $F_{Bx}$, and θ represents the angle between the geometric center connection line and the flexure hinge horizontal line.

The input displacement $x_{in}$ and output displacement $x_{out}$ of the bridge mechanism can be deduced and expressed as [23]:

(3) $$\left(\begin{array}{c} x_{in}\\ x_{out} \end{array}\right)=\left[\begin{array}{cc} c_{11} & c_{12}\\ c_{21} & c_{22} \end{array}\right]\times \left(\begin{array}{c} F_{in}\\ F_{out} \end{array}\right)$$

where $c_{11}$, $c_{12}$, $c_{21}$, and $c_{22}$ are the elements of the flexibility matrix of the bridge mechanism, which can be expressed as:

(4) $$c_{11}=\frac{2\cos^{2}\theta }{k_{l1}}+\frac{\cos^{2}\theta }{k_{l2}}+\frac{sin^{2}\theta \left(3l^{2}-6ll_{1}+4l_{1}^{2}\right)}{6k_{\theta 1}}+\frac{\sin^{2}\theta \left(3l^{2}-6ll_{2}+4l_{2}^{2}\right)}{12k_{\theta 2}}$$

(5) $$c_{12}=\frac{\sin\theta \;\cos\theta \left(3l^{2}-6ll_{1}+4l_{1}^{2}\right)}{6k_{\theta 1}}+\frac{\sin\theta \;\cos\theta \left(3l^{2}-6ll_{2}+4l_{2}^{2}\right)}{12k_{\theta 2}}\;-\frac{2\sin\theta \;\cos\theta }{k_{l1}}-\frac{\sin\theta \;\cos\theta }{k_{l2}}$$

(6) $$c_{21}=\frac{\sin\theta \;\cos\theta \left(3l^{2}-6ll_{1}+4l_{1}^{2}\right)}{6k_{\theta 1}}+\frac{\sin\theta \;\cos\theta \left(3l^{2}-6ll_{2}+4l_{2}^{2}\right)}{12k_{\theta 2}}\;-\frac{2\sin\theta \;\cos\theta }{k_{l1}}-\frac{\sin\theta \;\cos\theta }{k_{l2}}$$

(7) $$c_{22}=\frac{2\sin^{2}\theta }{k_{l1}}+\frac{\sin^{2}\theta }{k_{l2}}+\frac{cos^{2}\left(3l^{2}-6ll_{1}+4l_{1}^{2}\right)}{6k_{\theta 1}}+\frac{\cos^{2}\theta \left(3l^{2}-6ll_{2}+4l_{2}^{2}\right)}{12k_{\theta 2}}$$

where *l* is the length of the geometric center connecting the line of the flexure hinge, expressed as:

(8) $$l=\sqrt{{\left(l_{1}+l_{2}\right)}^{2}+{\left(l_{1}+l_{2}\right)}^{2}\tan^{2}\theta }$$

where $k_{l1}$, $k_{l2}$, $k_{\theta 1}$, and $k_{\theta 2}$ are, respectively, the tensile stiffness, the rotational stiffness of the flexible hinge, and the rectangular bar of the bridge mechanism.

(9) $$\left\{\begin{array}{cc} k_{l1}=\frac{Ebt}{l_{1}} & k_{l2}=\frac{Ebt}{l_{1}}\\ k_{\theta 1}=\frac{Ebt^{3}}{12l_{1}} & k_{\theta 2}=\frac{Ebh^{3}}{12l_{2}} \end{array}\right\}$$

## 3. Optimal Design of the Bridge Mechanism

**Figure 3:**
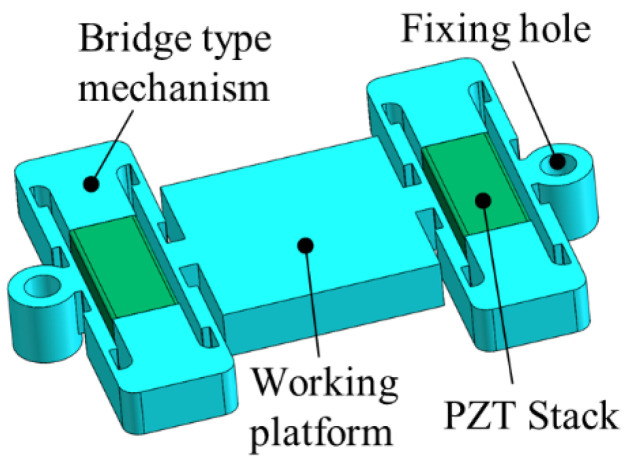
The series bridge PZT actuator.

According to the output characteristics of the classical bridge mechanism, a series bridge PZT actuator of the “simply supported beam-type” was optimized and designed to reduce the nonlinear error of the actuator displacement curve and improve the natural frequency of the actuator, as shown in Figure 3. The structure of the actuator is composed of a working platform and two bridge mechanisms in series, and the fixed holes are distributed outside the bridge mechanism. The two bridge mechanisms drive the working platform alternately, and both the push stroke and the return stroke are actively driven, which reduces the nonlinear error of the actuator output displacement. Compared with the classical bridge mechanism with unilateral constraints, the series bridge mechanism with bilateral constraints has a higher natural frequency.

### 3.1. Parametric Analysis of the Series Bridge Mechanism

The displacement magnification directly determines the driving stroke of the series bridge PZT actuator [24], and the correlation between the structural parameters and the displacement magnification should be considered. In addition, when the PZT stack is under external load, the actual maximum output displacement is less than that without load; thus, the influence of input stiffness of the bridge mechanism should be considered. When $F_{out}=0$ as the output force of the actuator, the following equation can be deduced according to Equation (3).

(10) $$\lambda =\frac{x_{out}}{x_{in}}=\frac{c_{21}}{c_{11}}$$

(11) $$k_{in}=\frac{F_{in}}{x_{in}}=\frac{1}{c_{11}}$$

The displacement magnification λ and the input stiffness $k_{in}$ can be expressed as follows:

(12) $$\lambda =\frac{\gamma k_{l1}k_{l2}tan\theta -\delta tan\theta }{\delta +\gamma k_{l1}k_{l2}\tan^{2}\theta }$$

(13) $$k_{in}=\frac{12k_{l1}k_{l2}k_{\theta 1}k_{\theta 2}}{\delta \cos^{2}\theta +\gamma k_{l1}k_{l2}sin^{2}\theta }$$

where $\gamma =2k_{\theta 2}\left(3l^{2}-6ll_{1}+4l_{1}^{2}\right)+k_{\theta 1}\left(3l^{2}-6ll_{2}+4l_{2}^{2}\right)$ and $\delta =12k_{\theta 1}k_{\theta 2}\left(k_{l1}+2k_{l2}\right)$.

Therefore, the displacement magnification λ and the input stiffness $k_{in}$ of the bridge mechanism are determined by the thickness of the bridge mechanism b, the length $l_{1}$ and the thickness t of the flexure hinge , the length of the rectangular rod $l_{2}$, the thickness of the flexible rod h, and the angle θ between the geometric center of the flexure hinge and the horizontal line. Taking the initial values $l_{1}=5\;\mathrm{mm}$, $l_{2}=10\;\mathrm{mm}$, t=1 mm,θ=5°,h=5 mm, and b=6 mm, the control variable method was used to analyze each structural parameter one by one.

**Figure 4:**
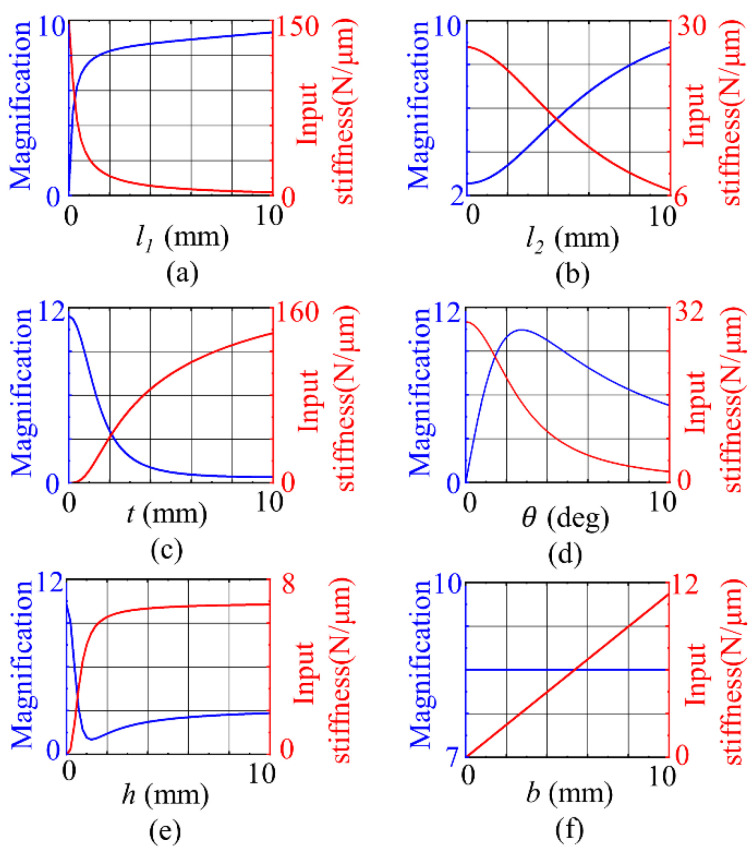
Influence of structural parameters on displacement magnification and input stiffness: (**a**) length of flexure hinge; (**b**) length of rectangular rod; (**c**) thickness of flexure hinge; (**d**) inclination of the line joining the geometric centers of the two flexible hinges; (**e**) thickness of rectangular rod; (**f**) thickness of the bridge mechanism.

As shown in Figure 4a,b, as the length of the flexible hinge and rectangular bar increases, the displacement magnification of the bridge mechanism increases and the input stiffness decreases accordingly. According to Figure 4c,e,f, the input stiffness of the bridge mechanism increases with the increase in the thickness of the flexible hinge, the thickness of the rectangular bar and the thickness of the bridge mechanism. The displacement magnification decreases with the increase in the thickness of the flexure hinge and decreases first and then increases with the increase in the thickness of the rectangular bar, which is independent of the thickness of the bridge mechanism.

### 3.2. Parameter Sensitivity Analysis

**Figure 5:**
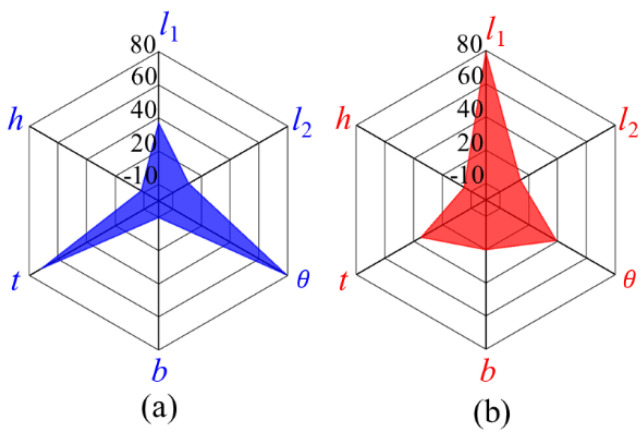
Parameter sensitivity analysis: (**a**) sensitivity of the displacement magnification; (**b**) sensitivity of the input stiffness.

The sensitivity of the structural parameters to the performance of the bridge mechanism was studied to provide a basis for the selection of parameter values. The machining accuracy of sensitive parameters was improved to reduce the influence of machining error on the performance of the actuator. The sensitivity analysis of displacement magnification and input stiffness was carried out to determine the dominant parameters affecting the performance of the bridge mechanism, as presented in Figure 5. The displacement magnification is more sensitive to the variation in the thickness of the flexure hinge *t* and the inclination angle of the central connection line *θ* than other parameters, and the input stiffness is more sensitive to the variation in the length of the flexure hinge $\;l_{1}$ than to other parameters.

### 3.3. Selecting the Parameters of the Series Bridge Mechanism

According to the above parameter analysis and parameter sensitivity analysis, and considering the properties of the material and the actual processing capacity, the appropriate structural parameters were selected and are listed in Table 1. The physical parameters of 7075 aluminum alloy are also provided in the table. Considering that the sensitivity of the thickness of the rectangular bar h to the displacement magnification and the input stiffness is very small, to reduce the processing difficulty, the upper and lower surfaces of the rectangular bar are coplanar with the sides of the flexible hinges on both sides, then the calculation formula of the thickness of the rectangular bar is as follows:

(14) $$h=\left(l_{1}+l_{2}\right)tan\theta +t$$

The displacement magnification λ=7.36 and $k_{in}=10.58\;N/\mu m$ are obtained by substituting the structural parameters into the calculation formulas of displacement magnification and input stiffness.

Due to the influence of the input stiffness of the bridge mechanism, the actual driving stroke of the PZT stack is less than the driving stroke $x_{m}$ without load. After amplification by the bridge mechanism, the theoretical maximum driving stroke is:

(15) $$x_{out_{max}}=\;\lambda \left(\frac{k_{PZT}}{k_{PZT}+k_{in}}\right)x_{m}$$

where $k_{PZT}$ is the stiffness of the PZT stack.

The dimensions of the PZT stack are 6×6×18 mm. Without load, the maximum drive stroke is 20 μm, and the stiffness is 60 N/μm. By substituting into the above equation, the theoretical maximum driving stroke $x_{out_{max}}$ of the bridge mechanism is 125.17 μm.

## 4. Results of the Finite Element Method

**Figure 6:**
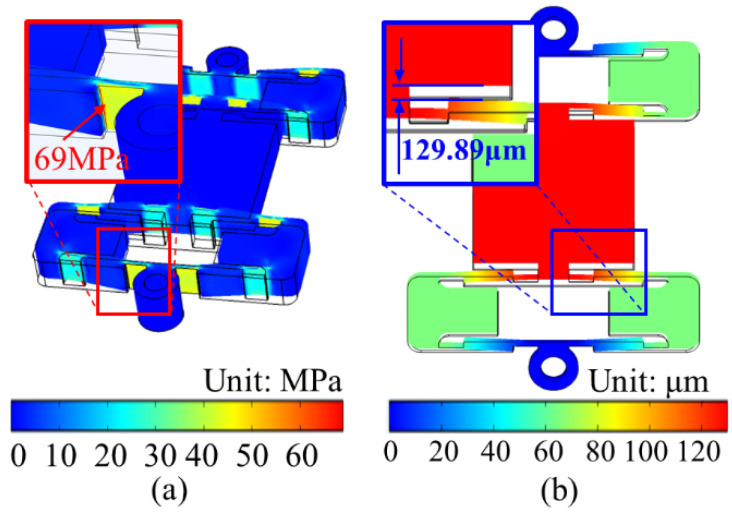
Static analysis of the series bridge mechanism: (**a**) stress analysis; (**b**) displacement analysis.

Using COMSOL Multiphysics 5.5 software (COMSOL Corporation, Stockholm, Sweden), the static and dynamic analysis of the series bridge mechanism was carried out to verify the rationality of the structure parameters. As the material of the series bridge mechanism, 7075 aluminum was selected and two fixed holes were set as fixed constraints. When a voltage was applied to the piezoelectric ceramics to make an output displacement of 17 μm (equivalent to the maximum output displacement of the PZT stack affected by the input stiffness of the bridge mechanism), the deformation of each part was as displayed in Figure 6, and the output direction of the displacement of the working platform meets the requirement. The stress field of the series bridge mechanism is presented in Figure 6a. The maximum stress generated at the connection between the flexible hinge and the rectangular bar was 69 MPa, which is far less than the yield strength of 7075 aluminum of 455 MPa. The actuator can be determined to be in the linear flexible stage during the operation. The displacement field of the series bridge mechanism is shown in Figure 6b, the maximum driving stroke is 129.89 μm and the displacement magnification is 7.64, and the error of the theoretical model is 3.77% and 3.81%, respectively.

**Figure 7:**
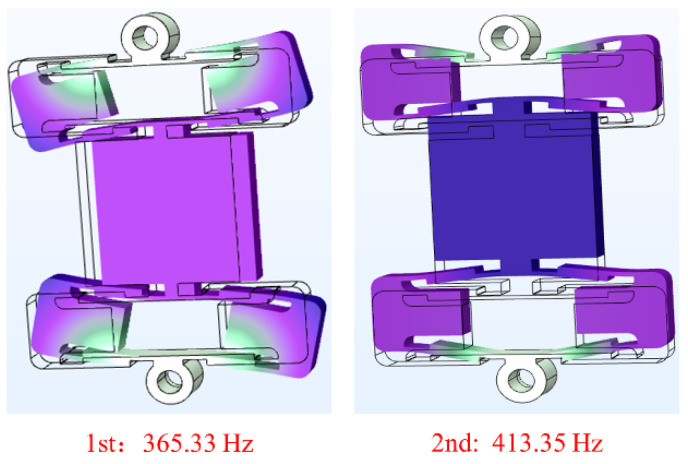
Characteristic frequency of the series bridge mechanism.

The operating frequency of a non-resonance PZT actuator needs to be lower than its first resonance frequency, so the characteristic frequency of the series bridge mechanism was simulated and analyzed. The fixing holes on the outside of the series bridge mechanism were set as fixed constraints, and the series bridge mechanism was meshed. The meshes contained 478,116 domain elements, 76,988 boundary elements, and 8780 edge elements. The first two resonance frequencies and the corresponding mode shapes of the series bridge mechanism are presented in Figure 7. The analysis results show that for the first mode shape of the series bridge mechanism, the working platform swings along the direction perpendicular to the main motion, and the corresponding resonance frequency is 365.33 Hz; for the second mode shape, the working platform moves along the main motion direction, and the corresponding resonance frequency is 413.35 Hz. The results of the characteristic frequency analysis show that the actuator should operate at a resonant frequency less than that of the first mode. The results of the characteristic frequency analysis show that the actuator should operate at a resonance frequency less than that of the first mode shape (365.33 Hz).

## 5. Experiments

**Figure 8:**
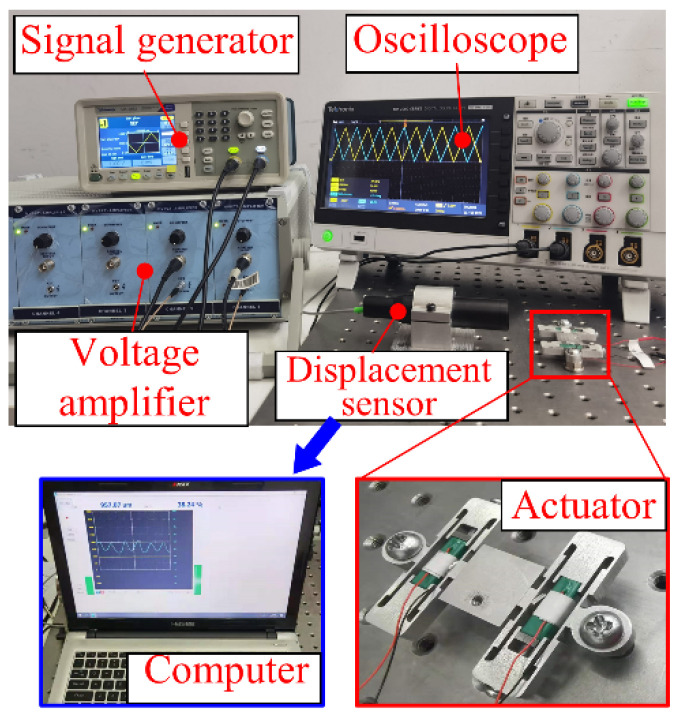
Experimental setup.

The experimental platform consisted of a two-dimensional piezoelectric positioning platform, a signal generator (Tektronix AFG1022, Tektronix Corporation, Beaverton, OR, USA), a power amplifier (core morrow E00. A4, Harbin, China), an oscilloscope (Tektronix TBS2000, Tektronix Corporation, Beaverton, OR, USA), a spectral confocal displacement sensor (Think Focus CDS-500, THINKFOCUS, Shanghai, China), and a computer. Two voltage signals were generated by the signal generator, which were amplified by the power amplifier and applied to the PZT stack. An oscilloscope was used to detect the waveform and amplitude of the voltage, and the spectral confocal displacement sensor was used to detect the driving displacement of the actuator, and the data were sent back to the computer. All components were placed on a vibration isolation table, as shown in Figure 8.

**Figure 9:**
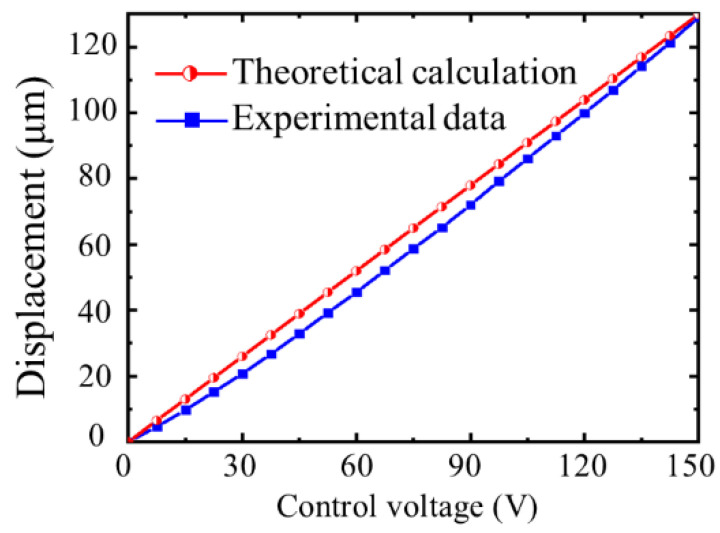
Maximum displacement of the actuator.

The maximum driving stroke of the actuator was tested and the displacement magnification was calculated. The experiment was carried out under open-loop control, and the test results are depicted in Figure 9. The maximum driving stroke of the actuator was 129.41 μm, the displacement magnification was 7.61, for an error of the theoretical model of 3.39% and 3.28%, respectively. There are two reasons for the discrepancy between the measured and calculated values. The first is the error due to processing and manufacturing. Secondly, in theoretical modeling, except for the flexure hinge and rectangular rod, other structures are regarded as rigid bodies and their tiny deformation is ignored.

**Figure 10:**
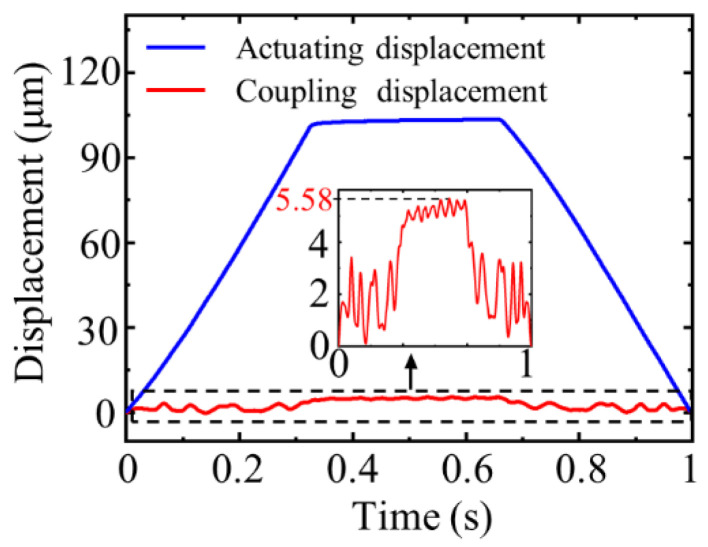
Non-driven displacement of the actuator.

A trapezoidal voltage signal with a slope of 360 V/s and a peak voltage of 120 V was applied to the PZT stack. The displacement curve of the driver is depicted in Figure 10. The driving stroke of the actuator is 103.23 μm, and the non-driven displacement is 5.58 μm. It is obvious from the figure that the non-driven displacement fluctuates greatly during the periods of voltage rise and fall because the symmetry of the bridge mechanism cannot be completely guaranteed under the actual machining conditions, making the output of the PZT stack different.

**Figure 11:**
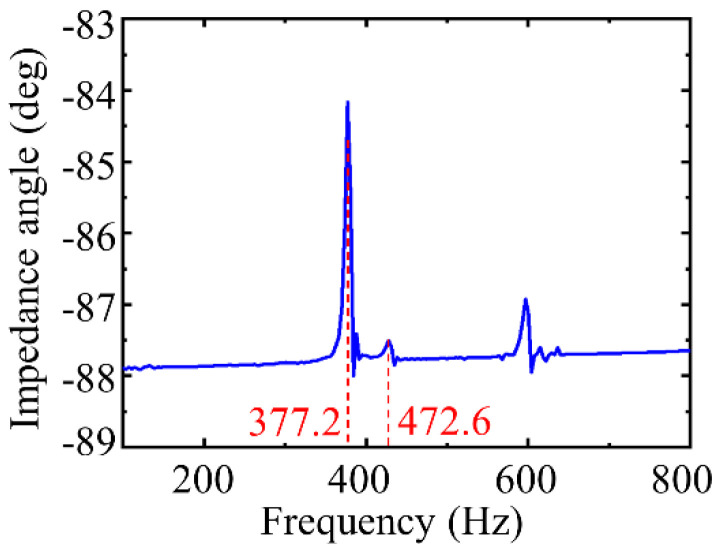
Frequency response of the actuator.

The resonance frequency of the actuator was measured by an impedance analyzer. By applying a sweep sinusoidal wave with an amplitude of 1 V and a frequency range of 100~800 Hz to the PZT stack, the curve between the impedance angle and the frequency was generated by impedance analyzer, as shown in Figure 11. The first two resonance frequencies of the actuator are 377.2 and 472.6 Hz, with errors of 3.15% and 12.54%, respectively, compared with the first two resonance frequencies calculated by the finite element method.

**Figure 12:**
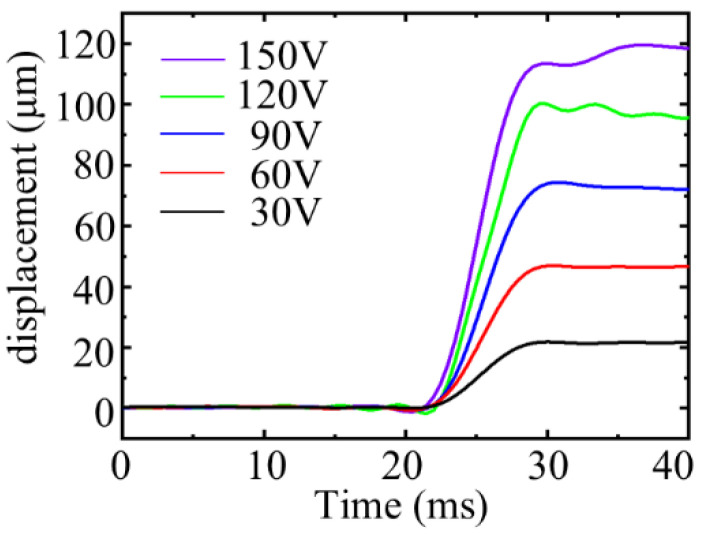
Response speed of the actuator.

Square wave signals of the same frequency and different amplitudes were applied to the PZT stack to test the response speed of the driver. After FFT low-pass filtering, the displacement curve is displayed in Figure 12. The response time of the actuator was kept within the range of 10 ms under different step voltages.

**Figure 13:**
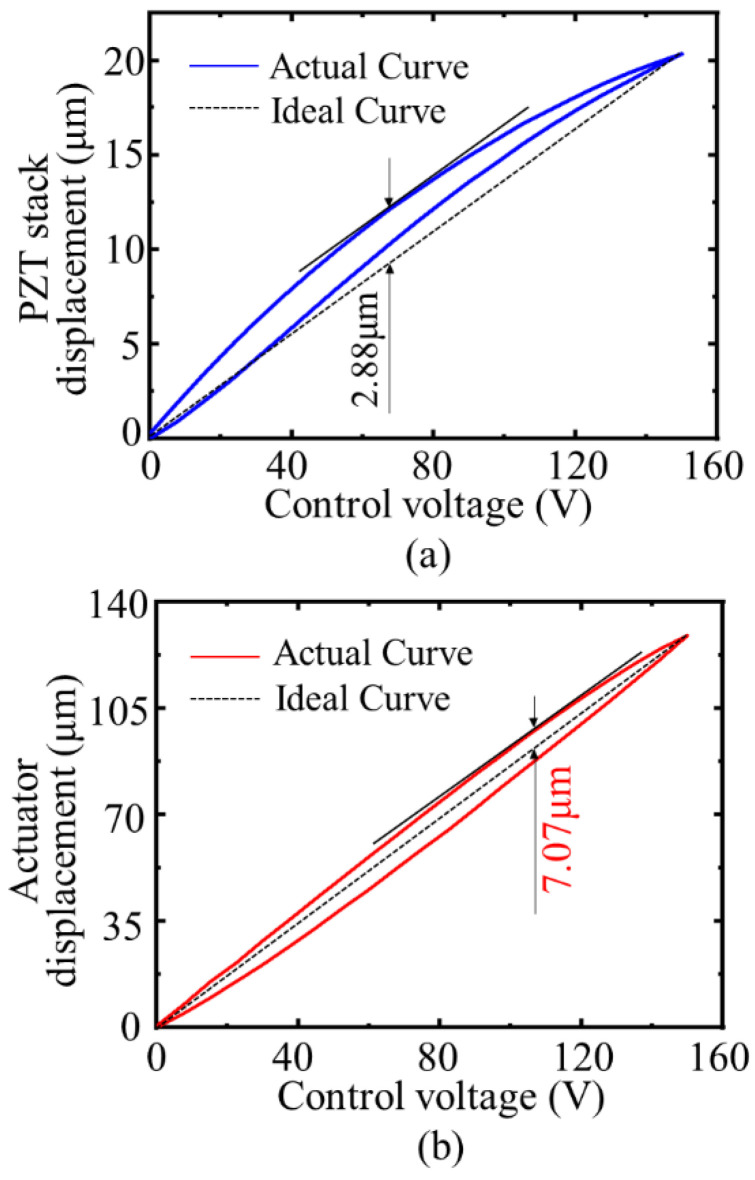
Hysteresis comparison: (**a**) hysteresis loop of the PZT stack; (**b**) hysteresis loop of the actuator.

The asymmetry of the displacement curve is the main factor that affects the positioning accuracy of the actuator. Comparing the hysteresis loops of the PZT stack and the actuator, the maximum displacement point and the zero displacement point of the actual displacement curve are connected to form an ideal curve. The ideal curve shows that the output displacement has a complete linear relationship with the control voltage. As depicted in Figure 13a, the maximum deviation between the hysteresis loop of the PZT stack and the ideal displacement curve is 2.88 μm and the nonlinear error is 14.41%. The hysteresis loop of the actuator is displayed in Figure 13b. The maximum deviation between the actual displacement curve and the ideal displacement curve is 7.07 μm, and the nonlinear error is 5.48%. The nonlinear error is reduced by 61.97%.

## 6. Conclusions

Aiming to overcome the shortcomings of the classical bridge mechanism in practical applications, a series bridge mechanism under double constraints was proposed in this paper. Based on the sensitivity analysis of the structural parameters of the bridge mechanism, a series bridge PZT actuator was optimized and designed. The performance parameters of the actuator, such as maximum stress, displacement magnification, and resonance frequency, were analyzed by finite element simulation. The experimental results show that the actuator has a maximum driving stroke of 129.41 μm with a high first-order resonance frequency (377.2 Hz), and the maximum nonlinear error of the hysteresis loop decreases from 14.41% to 5.48% of the PZT stack, a reduction of 61.79%. The proposed actuator realizes the bidirectional active drive between the push stroke and the return stroke, and effectively reduces the nonlinear error caused by the asymmetry of the output displacement.

The comparison of some of the parameters of the three existing actuators with those proposed in this paper is listed in Table 2.

1. Both the push stroke and return stroke are actively driven, rather than relying on the flexible effects of the structure.
2. With a higher natural frequency, the working range of the actuator is wider than that of other actuators.
3. The maximum nonlinear error of the actuator hysteresis loop is only 5.48%, and the maximum nonlinear error of the existing mechanism is between 10% and 15%.

## Figures and Tables

**Table 1:** Main parameters of the series bridge mechanism.

$l_{1}\;(\mathrm{mm})$	$l_{2}\;(\mathrm{mm})$	θ (deg)	*t* (mm)
4	8	5	0.8
***h* (mm)**	***b* (mm)**	**E (GPa)**	ρ **(kg/m^3^** **)**
1.85	6	71	2810

**Table 2:** Performance comparison of different actuators.

Reference	[11]	[25]	[26]	This Work
Dimension (mm^3^)	30 × 30 × 10	63 × 48 × 10	-	38 × 32 × 6
Resonance frequency (Hz)	190	130	178	377.2
Nonlinear error	14.22%	11.8%	10.63%	5.48%

## Data Availability

Data supporting the findings of this study are contained within the article.
